# GHS-OBAT: Global, open building attribute data reporting age, function, height and compactness at footprint level

**DOI:** 10.1016/j.dib.2025.111751

**Published:** 2025-06-06

**Authors:** Pietro Florio, Panagiotis Politis, Katarzyna Krasnodębska, Johannes H. Uhl, Michele Melchiorri, Ana M. Martinez, Georgia Kakoulaki, Martino Pesaresi, Thomas Kemper

**Affiliations:** a European Commission, Joint Research Centre (JRC), Ispra, Italy; b European Dynamics Belgium S.A., Brussels, Belgium; c Institute of Geography and Spatial Organization, Polish Academy of Sciences, Warsaw, Poland

**Keywords:** Building stock, Building age, Construction epoch, Building use, Building compactness, Building height, Vector-raster integration

## Abstract

Detailed geospatial data on building footprints represents a highly valuable source of information, as acknowledged by regulation in Europe. Such datasets are becoming increasingly available in Europe and at global scale, yet they often lack detailed and complete thematic information. Herein, we present the Global Human Settlement – Open Building Attribute Table (GHS-OBAT), a large-scale data integration effort to link thematic attributes to building geometries, namely 2.3 billion openly available building footprints from Overture maps. Specifically, we enrich building footprint geometries with information on the building construction epoch, building height, residential and non-residential building function, and building compactness, from open datasets in the Global Human Settlement Layer data suite. The footprint-level attributes in GHS-OBAT were extracted through vector-raster integration, by applying zonal statistics at the building feature level. The nearly-global coverage of these data adds new dimensions to large-scale building stock analyses, valuable for a wide range of application domains, including disaster risk management, studies of urbanization, energy transition and climate mitigation, affordable housing and renovation, urban design, or real estate.

**Table:** 

Abbreviations
ANBH: Average of the Net Building Height
BSO: EU Building Stock Observatory
BUTYPE: Built typology, a derived product of GHSL-BUILT-S, presented in [1]
CRS: Coordinate Reference System
EU: European Union (with EU27 being the European Union counting 27 member States)
EPSG: European Petroleum Survey Group (EPSG codes identify geographic coordinate reference systems univocally)
FUN: Functional use classification in the built-up domain, a derived dataset of GHS-BUILT-C
GADM: database of Global Administrative Areas
GADM L1: database of Global Administrative Areas, administrative subdivisions level 1
GHSL: Global Human Settlement Layer
GHS-AGE: Global Human Settlement Age, data released under GHSL (GHSL R2025A)
GHS-BUILT-C: Global Human Settlement Built-up Characteristics, data released under GHSL (GHSL R2023A)
GHS-BUILT-H: Global Human Settlement Building Height, data released under GHSL (GHSL R2023A)
GHS-BUILT-S: Global Human Settlement Built-up Surface, data released under GHSL (GHSL R2023A)
GHS-OBAT: Global Human Settlement – Open Building Attributes Table, data released under GHSL (GHSL R2024A)
LOD: Level Of Detail
MAE: Mean Absolute Error
MAPE: Mean Absolute Percentage Error
MEDAE: Median Absolute Error
OSM: Open Street Map
UTM: Universal Transverse Mercator projection
r: Pearson correlation coefficient
RES: residential functional use of buildings
NRES: non-residential functional use buildings
RMSE: Root Mean Squared Error

Specifications Table

**Table:** 

Subject	Geography
Specific subject area	Building stock geospatial database.
Type of data	GHS-OBAT (Global Human Settlement – Open Building Attribute Table) includes the following dataset distributions: • **GHS-OBAT_GLOBE_R2024A_CSV:** Attribute tables per country / administrative unit, with latitude and longitude of building footprint centroids: Comma Separated Value files (CSV) • **GHS-OBAT_GLOBE_R2024A_GPKG:** Attribute tables per country / administrative unit, with georeferenced building footprint centroids: GeoPackage files (GPKG) • **GHS-OBAT_COUNTRYSTATS_GLOBE_R2024A:** Country and sub-country level statistics: MS Excel files (XLSX) • Open Python code on GitHub: Python files (PY)
Data collection	We downloaded the Overture nearly-global open dataset of vector building footprints derived from the integration of several sources, from community-based ones to those leveraging on computer vision applied to satellite imagery; we enriched the data with attributes from the Global Human Settlement Layer (GHSL) through vector-raster integration carried out at building level, and extracted country and sub-country level statistics. Attributes include: building construction epoch, building height and building use type (residential / non-residential). Building shape factor (compactness indicator) was estimated using simple extrusion (LOD1), based on height derived from GHSL raster data, perimeter and area computed from Overture geometry.
Data source location	Source data was downloaded on 8 August 2024 from: 1. Overture Buildings release 2024-07-22.0 https://docs.overturemaps.org/release/2024-07-22.0/. 2. GHSL data release R2023 and R2025: ○ GHS-BUILT-H_ANBH R2023A: GHS Average of the Net Building Height at 100 m resolution, for the year 2018 10.2905/85005901-3A49-48DD-9D19-6261354F56FE ○ GHS-BUILT-C_FUN R2023: GHS Building Functional Classification at 10 m resolution, Residential / Non-residential, for the year 2018 10.2905/3C60DDF6-0586-4190-854B-F6AA0EDC2A30 ○ GHS-AGE R2025: Global gridded estimates of the dominant age of the built stock (1975–2020) 10.2905/d503bb56-9884-4e4d-bb8f-d86711d9f749 3. GADM database of Global Administrative Areas, version 4.1 https://gadm.org/download_world.html
Data accessibility	Repository name: GHS-OBAT R2024A - GHS Open Building Attribute Table at footprint level, with age, function and morphological information (2020). European Commission, Joint Research Centre (JRC) [Dataset] Data identification number: 10.2905/f41a22f1-5741-4c41-86eb-6384654f6927 Direct URL to data: http://data.europa.eu/89h/f41a22f1-5741-4c41-86eb-6384654f6927 Instructions for accessing this data: This dataset contains a package composed by 1) CSV attribute tables split by country according to GADM 4.1 [2] and by level 1 administrative areas (GADM L1) for large countries, 2) geospatial centroids of Overture Building footprints version 2024-07-22.0 with the attached attribute table split as before, 3) summary tables gathering attributes statistics by country. The original polygonal geometry of Overture Building footprints can be downloaded from the links in the data source section and it can be linked to this dataset through the unique GERS ID. The spatial delineation of GADM administrative units is available at GADM official website (accessible through the link in the data source section). The files are organized by country, with three-letter country codes defined in ISO 3166-1 and names of GADM L1 units for large countries split in sub-country territorial units. Code to generate this data: https://code.europa.eu/jrc-ghsl/building-data-integrator

## 1 Value of the Data

• Under the *High-Value Datasets Implementing Regulation* (Regulation (EU) 2023/138 [3]), building data is recognized as essential for transparency, innovation, and public policy. It supports urban planning, energy efficiency, climate resilience, and real estate markets by ensuring open access to geospatial and environmental data related to the built environment.
• Global open building footprint datasets typically lack reliable and consistent thematic attributes covering key indicators of the building stock, which are provided here.
• Our data addresses this gap by enriching 2.3 billion open building footprint polygons from Overture with remotely sensed thematic attributes derived from the open GHSL data (see data source location for more information).
• The provided attributes, i.e. estimates of the building construction epoch, of building height, building functional use type (residential / non-residential), and building shape factor provide useful insights for energy planning, in view of an energy transition towards renewables, and for assessing the impact of the built environment on climate change.
• The integrated dataset is relevant, among other domains, for disaster risk management, to improve the assessment of the building stock vulnerability and the allocation of resources in case of an emergency.
• Policy makers, urban planners and designers, demographers, educators and urban geographers benefit from this dataset, which provides insights on the temporal evolution of the building stock and the geographic distribution of the functional use of buildings (residential / non-residential), enabling studies of urban morphology including the vertical and temporal dimensions.

## 2 Background

Building footprint vector polygons are commonly used by architects, urban designers, city planners, and practitioners (in this paper, we refer to “building footprints” as the polygonal shapes of buildings on the ground, encompassing both roof and ground-level projections). They are becoming gradually popular among policy makers and analysts, due to their fine spatial detail and the ability to store and query object-based attributes, making them ideal for multi-thematic analyses. Their relevance is confirmed by the European Union legislation [3], which mandates free access to *High-Value Datasets*, including geospatial building data, in the broader framework of *Common European Data Spaces* [4].

Building footprint vector datasets at global scale are increasingly available (e.g. OpenStreetMap [5], Overture [6], Microsoft Global Machine Learning Buildings [7], and significant collections at regional scale like Google Open Buildings [8], EUBUCCO [9], OpenStreetMap-based regional releases [10,11], collections of cadastral data [12]). However, they often include only vector geometries with limited, or incomplete thematic attributes (e.g. the building height where available). Other open data initiatives, such as GHSL [1,13], produce consistent, multi-temporal gridded raster data of global coverage (see details in the data source location), which are useful for large-scale analyses and visualization purposes, but lack fine spatial detail at the building level.

Thus, the integration of vector data on building footprints and gridded, but thematically rich information on human settlements obtained from efforts based on remote-sensing greatly enhances the usefulness of building footprint data and opens new analytical avenues in building-stock related research, especially in transdisciplinary analyses across multiple thematic attributes.

## 3 Data Description

**Fig. 1:**
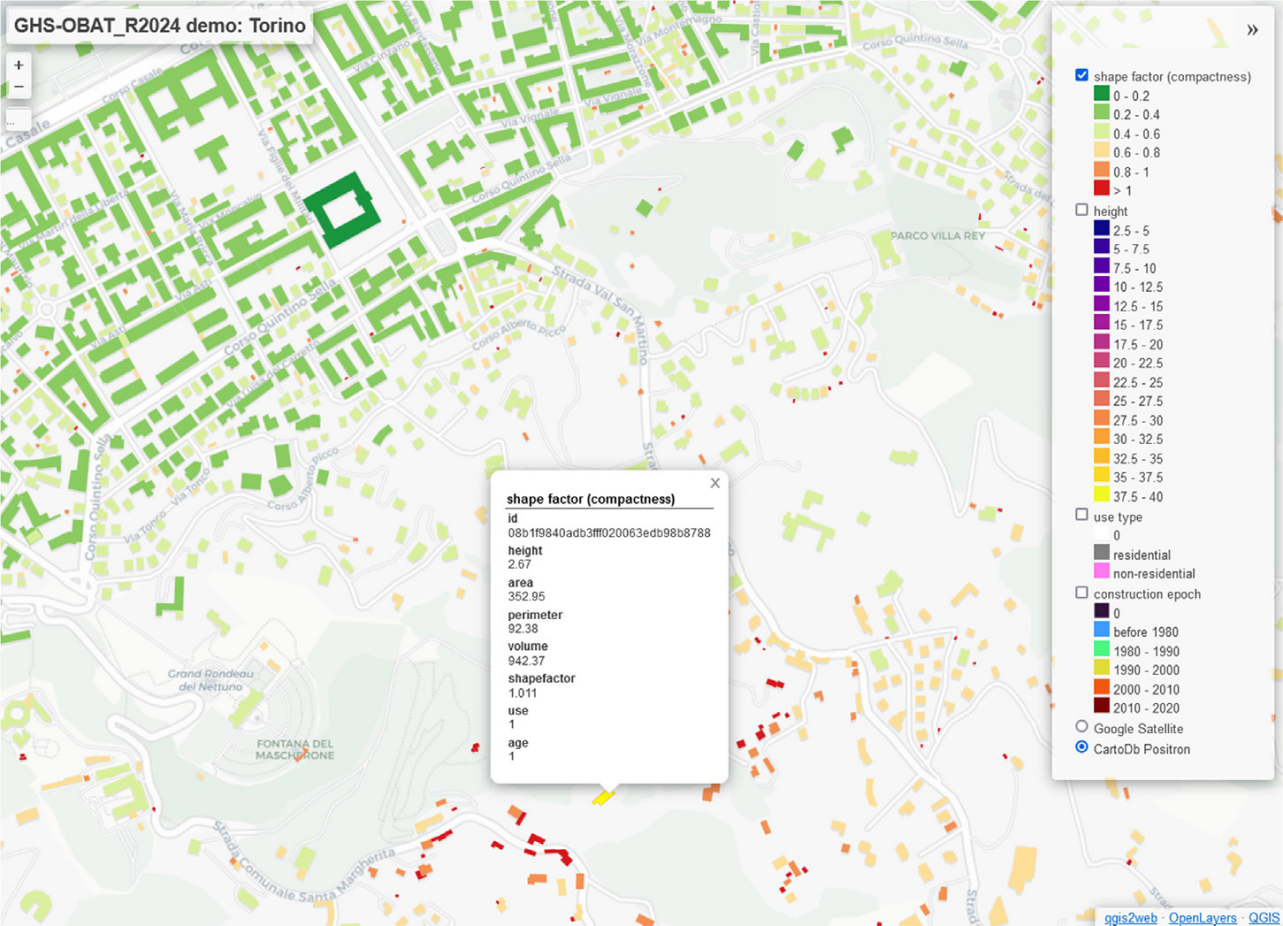
Example of a possible web-interface for the GHS-OBAT dataset in association with building footprint geometries from Overture, featuring the city of Torino (Italy).

**Fig. 2:**
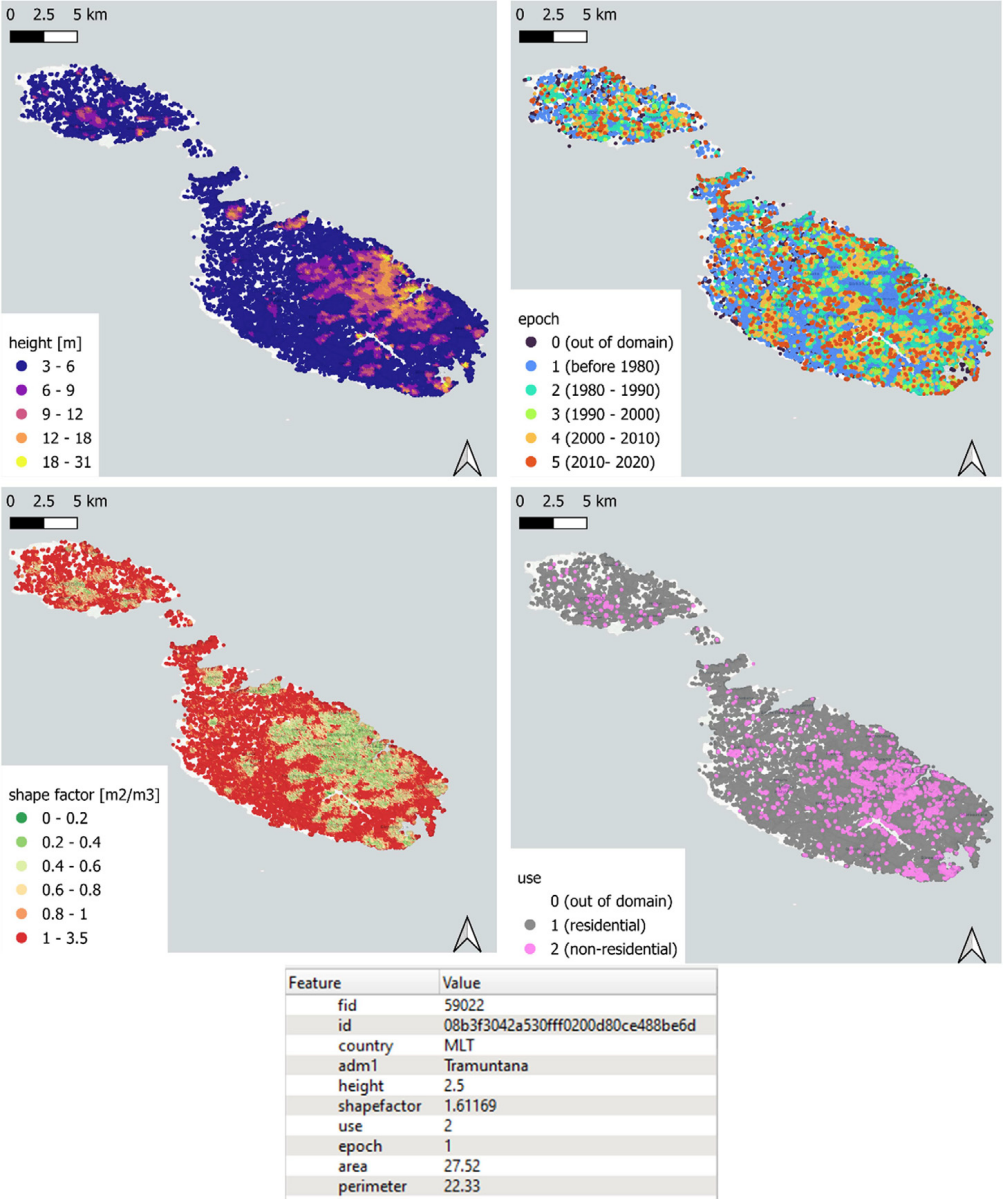
Preview of the GHS-OBAT dataset for the country of Malta (MLT) in a GIS environment. The four main attributes are represented, namely height, construction epoch, shape factor and use. At the bottom, the attributes of an example record are shown.

The main component of the dataset consists of an attribute table in CSV format split by country (and by GADM L1 for larger countries), each file with a maximum of circa 50 million building footprint features as rows and attributes as columns. Such table incorporates unique identifiers to link each set of attributes to the correct footprint polygon in the original geometry files (Fig. 1). The original geometry files can be downloaded from the Overture website linked in the “Data source location” section, following the instructions provided there. The original footprint geometries in polygonal format are not included in this data release, to avoid duplication of information available from Overture and save data storage space: as a consequence, users who want to work with the attributes illustrated here, in association with footprint polygons, must download the latter from Overture and link them to the attribute table through the provided unique identifier. Alternatively, the GHS-OBAT dataset is also released in GeoPackage format that incorporate the centroids of building footprints as geometry features, holding the same attributes of the CSV files, but much handier to use for further geospatial analyses (Fig. 2).

The attributes are derived from the GHSL R2023 data package [1,13], an open set of spatial raster datasets depicting settlements and population worldwide, including the distribution, age, and other characteristics of built-up areas; and from the GHSL R2025 GHS-AGE dataset [14]. A description of the attributes included in the table follows herewith:

• **id**: [string]. Unique identifier that constitutes the stable link to the geometry in Overture (the so-called GERS ID^1^). The Global Entity Reference System (GERS) is a framework for structuring, encoding, and matching map data to a shared universal reference. All features in Overture have a unique ID, a mechanism to match features across datasets, track data stability, and detect errors in the data. It is a 128-bit string of 32 hexadecimal characters.
• **lon:** [decimal]. The X-coordinate (longitude) of the centroid of the building footprint in WGS84 coordinate system (EPSG:4326), expressed in decimal degrees.
• **lat:** [decimal]. The Y-coordinate (latitude) of the centroid of the building footprint in WGS84 coordinate system (EPSG:4326), expressed in decimal degrees.
• **country:** [string]. ISO 3166-1 alpha-3 three-letter country code of the country the building footprint belongs to, according to an adjusted version of GADM 4.1 [2], made to fill gaps and overlaps and fix most unassigned territories. Building footprints not contained in any GADM level 0 country entity are marked as “XXX”.
• **adm1:** [string]. Text string holding the name of the GADM L1 unit. Building footprints not contained in any GADM L1 entity are marked as “XXX”.
• **height**: [decimal]. Mean building height calculated from the 100-m grid cells of the Average of the Net Building Height (ANBH) raster from the GHS-BUILT-H dataset [1,15], using zonal statistics. The ExactExtract library^2^ computes the mean height of the grid cells intersecting with each building footprint polygon, excluding any NoData values (e.g. value 0 in the ANBH raster). The height is expressed as floating-point number in meters, with a lower limit of 2.5 m. The ANBH raster is generated by extracting building height information from a composite of global Digital Elevation Models: the optical-based Advanced Land Observing Satellite World 3D [16] and the radar-based Shuttle Radar Topography Mission SRTM30 [17], both in ∼30 m resolution, and updating the building height estimations to year 2018 using shadow markers derived from Sentinel-2 imagery composite [18].
• **shapefactor**: [decimal]. Building shape factor: S/V [19], with S being the building surface (summing the roof, the floor and the façades) and V the volume of the building. This value is often correlated with the energy use for space heating or cooling of the building [19], therefore it is included here as one possible estimator of its energy efficiency. The shape-factor is estimated at feature level, approximating the building solid at LOD1 (Level Of Detail 1: simple extrusion, i.e. “shoebox”). The unit is m^2^/m^3^ (1/m) and it is inversely proportional to the building size.
• **use**: [integer]. Building functional use (residential / non-residential) derived from the mode (majority) of the intersecting 10-m resolution raster functional classification of the built domain (FUN) from the GHS-BUILT-C dataset [20]. The valid spatial domain for classification from GHS-BUILT-C is further extended by incorporating the 100-m functional classification from the BUTYPE raster grid, a derived product of the GHSL R2023A dataset [1] not included in the public release, available upon request to the authors. Building functional use is expressed as an integer number, with value 0 for footprints outside of the built-up domain (defined as the union of GHS-BUILT-C and BUTYPE), 1 for “residential” and 2 for “non-residential” built-up surface. The definition of residential built-up areas is aligned to the INSPIRE definition^3^ and includes areas dedicated prevalently for residential use, including mixed with other non-conflicting uses. The non-residential areas are dedicated exclusively to non-residential use, and predicted by observation of radiometric, textural and morphological features derived from Sentinel-2 satellite imagery [1].
• **epoch:** [integer]. The building construction year is derived using the mode (majority rule) of the intersecting 100-m grid cells from the GHS-AGE raster, representing global gridded estimates of the dominant age of the built stock for the period 1975–2020 [14], a derived product of the GHS-BUILT-S dataset [21,22]. Specifically, the spatio-temporal data cube of the GHS-BUILT-S dataset, which measures sub-pixel built-up surface at 100 m resolution globally from 1975 to 2020, was queried along the temporal domain, to identify the year in which at least 50 % of the built-up surface in 2020 was exceeded in each grid cell. This year represents the most likely construction epoch of the built stock within each grid cell, in the absence of more detailed building-level construction year information [14]. The estimated building construction epochs range between 1980 and 2020, with a time step of 10 years: it is expressed as an integer number ranging from 1 to 5 (respectively before 1980, 1980–1990, 1990–2000, 2000–2010, 2010–2020), with 0 being footprints outside of the built-up domain of GHS-BUILT-S.
• **area**: [decimal]. Surface of the Overture building footprint, expressed as floating-point number in meters squared, computed in the local UTM projection of the building footprint.
• **perimeter**: [decimal]. Perimeter of the Overture building footprint, expressed as floating-point number in meters, computed in the local UTM projection of the building footprint.

In addition to the attribute table in CSV or GeoPackage format, summary statistics for the dataset attributes are distributed at country and GADM L1 level (see Table 1), to ease cross-country comparative analyses.

**Table 1:** Summary statistics available at country and GADM L1 level for the attributes contained in the GHS-OBAT dataset.

Summary field	Suffixes	Unit	Description
Surface_*	* = min, q1, median, average, q3, max, sum	m^2^	Building footprint surface aggregation in the country of interest (or GADM L1 unit): respectively minimum, 1^st^ quartile, median, average, 3^rd^ quartile, maximum, sum.
Height_*	* = min, q1, median, average, q3, max	m	Building height aggregation in the country of interest (or GADM L1 unit): respectively minimum, 1^st^ quartile, median, average, 3^rd^ quartile, maximum.
Shapefactor_*	* = min, q1, median, average, q3, max	m^2^/m^3^ (1/^m)^	Building shape factor aggregation in the country of interest (or GADM L1 unit): respectively minimum, 1^st^ quartile, median, average, 3^rd^ quartile, maximum.
Footprint_count_Use_*	* = 0, 1, 2	-	Number of building footprints in each of the functional use categories: respectively 1 for residential and 2 for non-residential. 0 is for building footprints outside the built-up raster domain.
Footprint_surface_ha_Use_*	* = 0, 1, 2	hectares	Building footprint surface sum in each of the functional use categories: respectively 1 for residential and 2 for non-residential. 0 is for footprints outside the built-up raster domain.
Footprint_count_Epoch_*	* = 0, 1, 2, 3, 4, 5	-	Number of building footprints in each of the construction epochs: respectively 1 for before 1980, 2 for 1980–1990, 3 for 1990–2000, 4 for 2000–2010, 5 for 2010–2020. 0 is for building footprints outside the built-up raster domain.
Footprint_surface_ha_Epoch_*	* = 0, 1, 2, 3, 4, 5	hectares	Building footprint surface sum in each of the construction epochs: respectively 1 for before 1980, 2 for 1980–1990, 3 for 1990–2000, 4 for 2000–2010, 5 for 2010–2020. 0 is for building footprints outside the GHS-BUILT-S raster domain.

## 4 Experimental Design, Materials and Methods

**Fig. 3:**
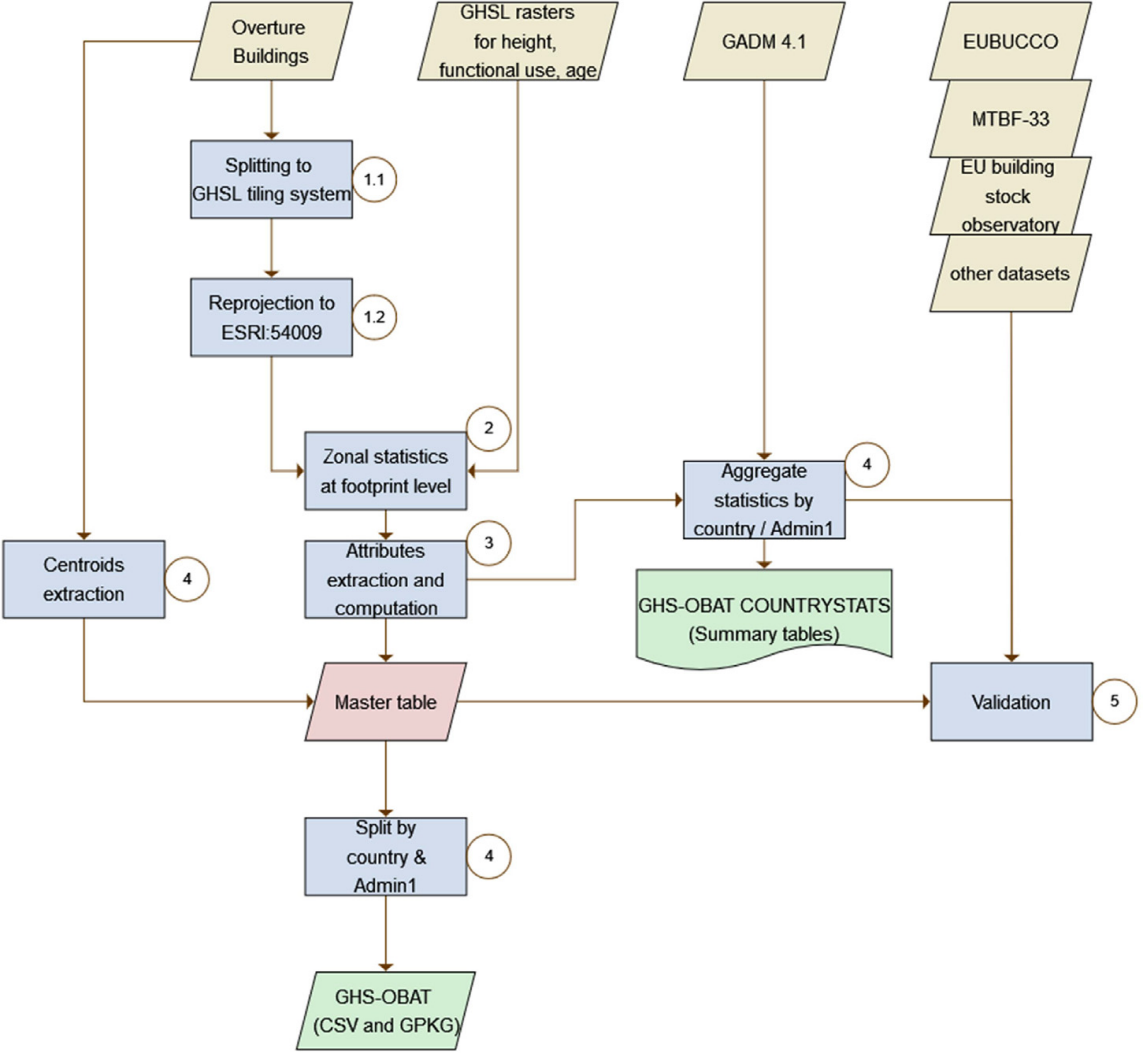
Illustration of the methodological workflow. Datasets are shown as parallelograms, statistics reports as a rectangle with wavy boundaries, processing steps as rectangles. Input data is in yellow, intermediate (unpublished) data is in red, output is in green. Blue color refers to processing steps, with a circled number pointing to the section describing each into detail. (For interpretation of the references to color in this figure legend, the reader is referred to the web version of this article.).

All the geospatial operations described below, and summarized in Fig. 3, were performed in Python v3.11, using widely adopted geospatial libraries: Geopandas, Rasterio, Shapely, pyproj, Exact Extract, duckdb with spatial extension, along with their respective dependencies.

**1. Pre-processing**

After downloading the datasets listed in the data source section, some pre-processing operations were carried out before the actual integration of building footprint vectors and raster grids.

**1.1. Splitting to a standard tiling system**

Building footprint vector sources use different tiling systems to split the data into lightweight, easily readable and manageable files; GHSL raster files are also split, yet into another tiling system, which uses a 25 × 25 km tile schema in World Mollweide projection (ESRI:54009). An alignment of the respective tiling systems is necessary for processing matched geographic areas: the GHSL tiling system was used for such purpose. The process starts by querying the Overture buildings based on the bounding box of a given GHSL tile, which is re-projected into WGS84. This ensures that the correct buildings are selected based on the extent of the GHSL tile in WGS84 coordinate system. After selection, the building footprints are split according to the GHSL tiling system, aligning them with raster data for consistency. This tiling system not only ensures alignment with raster files but also enables efficient processing and scalable analysis by supporting the iteration and parallelization of tasks.

**1.2. Alignment to a common Coordinate Reference System (CRS)**

After selecting the relevant buildings, the next step is to re-project the data into World Mollweide to align it with the GHSL tiling schema and further process the data.

**2. Raster-vector integration using zonal statistics**

Raster statistics were computed at the building footprint level using the ExactExtract library's specific functionality for calculating zonal statistics. This method calculates values for grid cells that intersect each building footprint, providing accurate results even when only partial grid cells are involved. In cases where a building footprint does not intersect any valid raster grid cells from the GHSL data, the attribute assigned to the building is marked as "out of domain", as discussed further in the limitations section: the number of out-of-domain buildings is reported in the validation section.

For building height, the mean height value was calculated using the GHS-BUILT-H dataset, based on the intersecting 100-m grid cells. The zonal statistics compute the mean height within each building footprint's boundary, considering the grid cells that intersect the footprint. As a result, neighboring buildings that are less than 100 m apart often inherit the same height value.

Similarly, for categorical raster grids, such as GHS-AGE (construction epoch) or GHS-BUILT-C and BUTYPE (building functional use), zonal statistics were used to determine the majority category within each footprint’s boundary. This process involves extracting the number of grid cells corresponding to each category (e.g., residential vs. non-residential) within the footprint’s boundary, ensuring that the building’s functional use and construction epoch are accurately attributed based on the most prevalent values within the footprint.

**3. Attributes assignment**

The country and GADM L1 attributes were assigned to building footprints by spatial join. Each building footprint was attributed with the mean building height of intersecting grid cells; the building construction epoch and the functional use class (residential / non-residential) were assigned based on the majority of grid cells within the footprint’s boundary.

The building shape factor (as an inverse proxy for compactness, Eq. 1) was calculated at feature level, approximating the ratio of the building outer surface (S) to the building volume (V) at LOD1 (simple extrusion, i.e. “shoebox”). With A footprint area, P footprint perimeter and h mean building height, the shape factor results in:

(1) $$shape\;factor=\frac{S}{V}=\frac{2A+P\cdot h}{A\cdot h}=\frac{2A}{A\cdot h}+\frac{P\cdot h}{A\cdot h}=\frac{2}{h}+\frac{P}{A}$$

Shape factor represents the inverse of compactness (Eq. 2), with higher building shape factor value indicating lower building compactness, and vice versa.

(2) $$compactness=\frac{V}{S}$$

**4. Splitting files and creating aggregated statistics**

The output master table was created by concatenating the tiled files produced in the previous steps. Centroids of building footprints were computed and extracted at this stage. The master table was then split by adjusted GADM country boundaries and by GADM L1 for larger countries, for each file to have a maximum in the order of circa 50 million building footprint features. Summary statistics were also calculated at country level (see Table 1) to describe the geospatial variability of attributes throughout the world.

**5. Validation**

Overall globally, the source GHSL raster grids largely match the spatial domain of Overture buildings, and they could generate attributes for the vast majority of footprints; except from 7 % of footprints for building heights and 4 % of footprints for functional use and construction epoch respectively, which fall out of the covered spatial domain (see the limitations section for possible reasons).

For validation purposes, we assessed the agreement of the estimated attributes mentioned above, i.e. building height, shape factor, functional use (residential / non-residential) and building construction epoch with authoritative attributes compiled in the EUBUCCO database [9] and other building footprint data. EUBUCCO is a conflated cadastral and Open Street Map (OSM)^4^ database of around 202 million building footprints across 28 European countries, attributed, where available, with values concerning height, construction epoch and functional use, here taken as a reference in the validation process. Moreover, we used building construction information from the Multitemporal Building Footprint Dataset for 33 US Countries (MTBF-33 [23]), and gridded building construction year information derived from proprietary U.S. real estate databases [24].

In the first validation step, we assessed the performance of the attribute integration process at feature level. For this purpose, we assigned GHSL raster attributes to the geometry of EUBUCCO and MTBF-33 building footprints originating from cadastral databases; we compared available attribute values for each building from the two sources, i.e. GHSL and authoritative cadastral sources used as reference. For continuous attributes like building height and its derivative shape factor, the following performance measures were computed: Root Mean Squared Error (RMSE), Mean Absolute Error (MAE), Median Absolute Error (MEDAE) and a Pearson correlation coefficient (r). For categorical variables such as building age class, we report Precision, Recall, and F-score.

In the second validation step, we assessed the accuracy of the building height, construction epoch and functional use at aggregated level (GADM L1 grouped by country). Cadastral sources in EUBUCCO for building heights and functional uses, as well as other reference data for construction epochs were considered for validation against GHS-OBAT. To ensure the comparability of data, for each thematic attribute we considered only the cadastral data compatible with the definition of remotely-sensed attributes from GHSL. For building height and functional use validation, we used data from GADM L1 units, where the total area of buildings in GHS-OBAT (A_p_) and the one in EUBUCCO (A_ref_) are comparable, i.e. their normalized difference over the whole territorial unit (Eq. 3) is between -0.25 and 0.25. In addition, the number of buildings by functional use and construction epoch per EU country was compared against information in the EU Building Stock Observatory database [25,26].

(3) $$normalized\;area\;difference=\;\frac{A_{p}-A_{ref}}{A_{p}+A_{ref}}$$

**5.1 *Height***

For height validation, we considered cadastral data from EUBUCCO (Table 2), in which the building height was estimated as distance from ground to the lowest point of the roof, to median/mean roof height, to eaves or to the 70th percentile of the point cloud generated during scanning: this information is available in the EUBUCCO metadata table^5^ listing the various data sources.

**Table 2:** Errors in building height estimation between GHS-OBAT and reference data from compatible cadastral sources in EUBUCCO. Mean Absolute Error (MAE), Mean Absolute Percentage Error (MAPE), Root Mean Square Error (RMSE), Median Absolute Error (MEDAE) and Pearson coefficient (r) are calculated by country, then weighted by the number of buildings shown in the righter most column to return the overall mean scores (in the last row).

Country	MAE	MAPE	RMSE	MEDAE	r	Number of buildings
CHE	4.32	280 %	5.18	3.93	0.26	2,640,847
DEU	2.88	95 %	4.05	2.31	0.31	19,367,651
FRA	2.68	61 %	3.59	2.14	0.33	43,120,207
ITA	3.71	88 %	4.93	3.02	0.28	5,205,389
LUX	2.74	47 %	3.33	2.50	0.35	143,923
NLD	2.91	77 %	3.80	2.43	0.20	9,692,657
OVERALL	**2.88**	**81%**	**3.89**	**2.72**	**0.29**	**80,170,674**

At building level, which involves the considerable bias introduced by oversampling the source raster to 10 m, the error could be quantified in MAE = 2.88 m for the height (Table 2), resulting in MAE = 0.44 m^2^/m^3^ (1/m) for its derived shape factor. It is worth noting that, by definition, ANBH raster providing building height information captures average heights over 100-m size pixels rather than height variability [15], i.e. it under detects high-rise peaks in building height: this explains the low association between estimate and reference from *r* Pearson coefficient in Table 2.

**Fig. 4:**
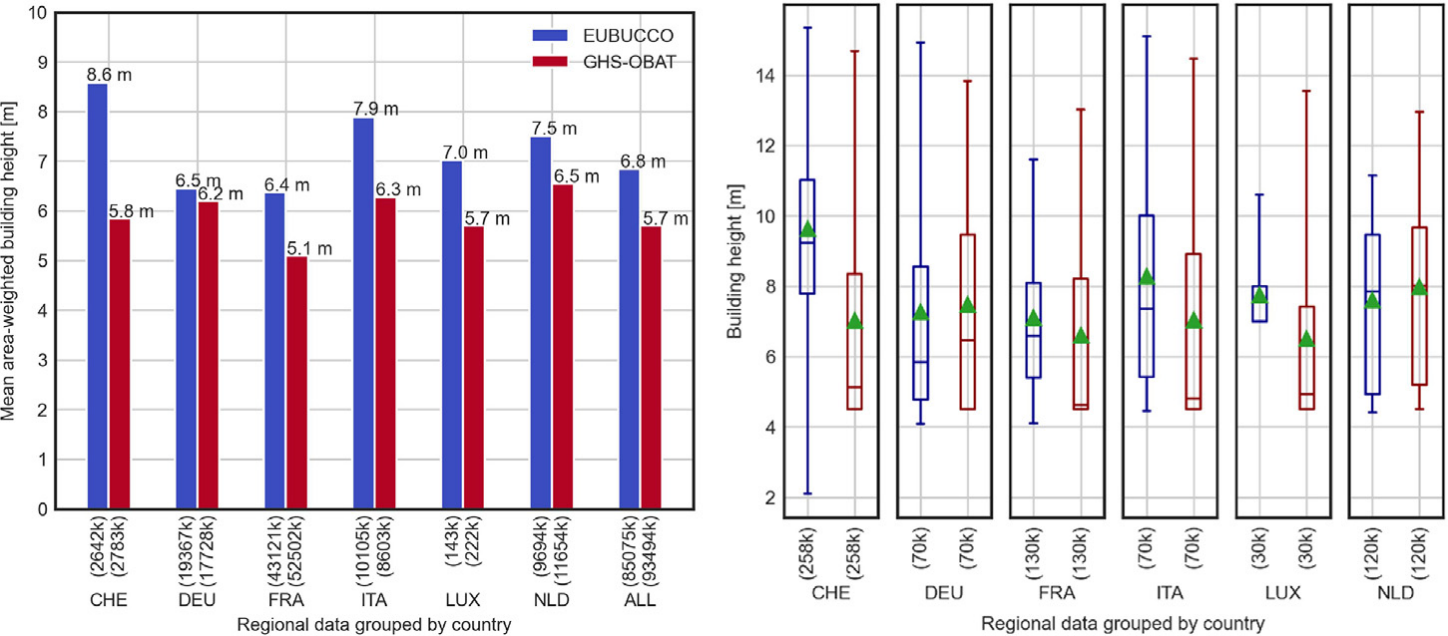
Left: Mean area-weighted building heights computed using cadastral data from EUBUCCO database and building footprints from GHS-OBAT database, grouped by country of origin, and for all buildings together. The number of buildings is shown in brackets below the bars. Right: Distribution of building heights for sampled data from EUBUCCO and GHS-OBAT databases. Mean value is shown with a triangle, median value is shown with a dash. Boxplot whiskers cover data range between 5th and 95th percentile of building heights. Data outside this range are not shown on the plot. (For interpretation of the references to color in this figure legend, the reader is referred to the web version of this article.).

Fig. 4 At an aggregated level, building height data from 144 out of 154 available GADM L1 units were comparable. Fig. 4 the mean building heights, weighted by building footprint areas, were computed using both cadastral information from EUBUCCO and using height data presented here (GHS-OBAT). The distribution of building heights (Fig. 4 – right panel) was generated for a subset of building footprints, randomly sampled from each GADM L1 unit, with sample size equal 10,000 building footprints per each GADM L1 unit (or the total amount of building footprints if lower than 10,000).

The comparison of the building heights shows an underestimation of the downscaled GHS-BUILT-H heights as compared to the cadastral data (Fig. 4). The difference between mean area-weighted building heights obtained for EUBUCCO and GHS-OBAT datasets varies substantially between investigated areas (Fig. 4, left panel), ranging from -2.7 m (in Switzerland) to -0.3 m (in Germany), and equals -1.1 m for all data evaluated together. Except for data collected in Switzerland and Luxembourg, the distributions of building heights (Fig. 4, right panel) are similar for both datasets. In all the cases, apart from the Netherlands and Germany, the median value of heights assigned to buildings is visibly lower for GHS-OBAT than for the cadastral data, and close to 2.5 m – the minimum building height assumed in GHS-BUILT-H layer. Overall, the difference in estimated area-weighted heights does not exceed the height of one building storey. A small error (-1.1 m) for all data, combined with the consistent, globally available definition of building height used in GHS-OBAT, confirm the usefulness of the validated attribute values for analyses involving averaged building height information, implemented at a large-scale.

**5.2 Functional use**

**Fig. 5:**
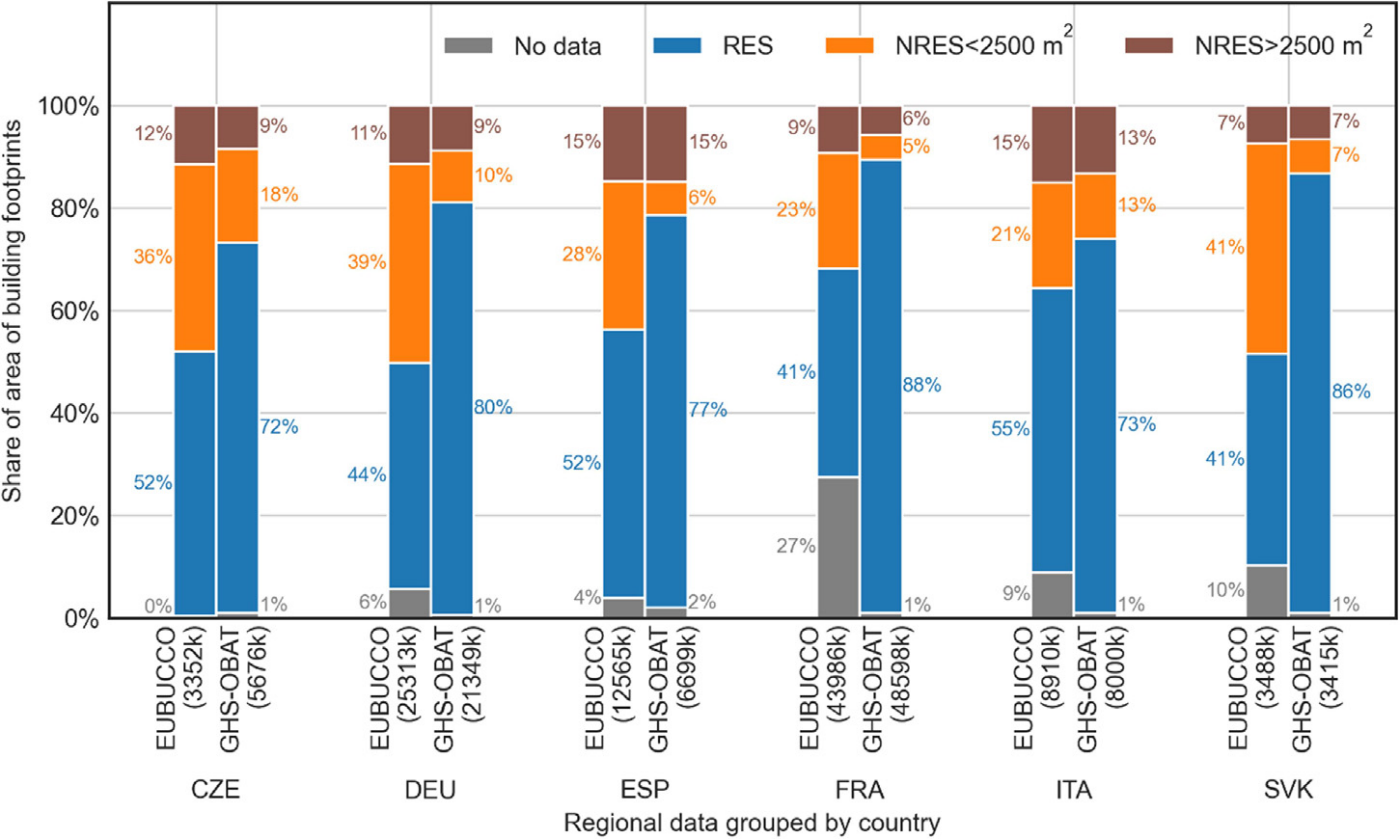
Share of area of building footprints with residential (RES), non-residential below or above 2500 m^2^ of footprint area (NRES<2500 m^2^ and NRES>2500 m^2^ respectively) or not assigned (No data) functional use classification computed using selected cadastral data from EUBUCCO database and the GHS-OBAT building footprints with downscaled GHS-BUILT-C data, grouped by country of origin. The number of buildings is shown in parentheses below the bars.

To validate functional use attributes, we used those cadastral data from EUBUCCO whose definition of functional use is compliant with that used in the GHSL data, where the non-residential classification is allocated to exclusive non-residential use. There are 84 GADM L1 units with compliant definitions: at feature level in such units, the assignment of the functional use performs well, with a Precision of 70 %, a Recall of 99 % and an F1 score of 82 %; this means commission error is higher than omission error. Among the 84 GADM L1 units, 56 were comparable with the reference data in terms of area of building footprints and were used for further validation. For cadastral data from the EUBUCCO dataset and for GHS-OBAT data, we calculated the shares of the total building areas with assigned residential (RES), non-residential (NRES) and unassigned (No data) uses, in the total building area at country level (Fig. 5). In GHSL data, the functional use is determined through remote sensing features, including the size of the building footprint: such features drive the non-residential classification. To identify the impact of building footprint size on the accuracy of the non-residential use assignment, we calculated the compared shares of non-residential building footprints with an area of *less than* vs *more than* 2500 m^2^: this is a proxy for the minimum roof size of 50 × 50 m, used as one of the criteria in the GHSL non-residential use attribution [1].

The results of the functional use validation show a high compliance of the non-residential use allocation to building footprints larger than 2500 m^2^ for GHS-OBAT against EUBUCCO, with a difference in the shares of building areas not exceeding 3 % at country level (Fig. 5, brown bars). The allocation of residential use strongly differs between EUBUCCO and GHS-OBAT data, possibly due to the difference in the functional use definitions utilized in GHSL data and among different authoritative sources, particularly for smaller buildings (<2500 m^2^). The downscaling of GHS-BUILT-C data results in a functional use attribute assignment on building footprint level, which is complete (share of building area with no functional use assigned is not exceeding 2 %), homogenous (functional use definition is independent of local definitions, varying between countries) and reliable (non-residential building area is not exceeding 27 %, as opposed to cadastral data, where non-residential building area is close to 50 % in data collected in Czech Republic or Slovakia).

In the data reported by the EU Building Stock Observatory [25], the total number of buildings per country in EU27 is broken down by functional use, including some categories for residential (i.e. apartment buildings, multi-family housing, single-family housing) and non-residential (i.e. educational, health, hotels and restaurants, offices, commercial and other non-residential buildings).

**Fig. 6:**
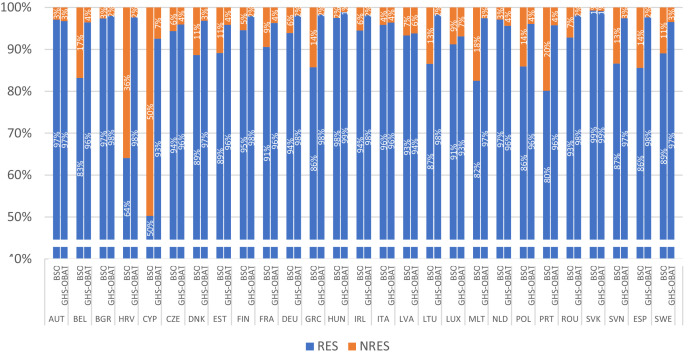
EU Building Stock Observatory (BSO) vs GHS-OBAT comparison of the number of buildings in each functional use class (RES / NRES), by country.

In a comparison against the EU Building Stock Observatory, GHS-OBAT underestimates the number of buildings in the non-residential class in some countries (Fig. 6). However, only in circa one third of the countries in EU27 (10/27) the difference exceeds 10 % of non-residential buildings; it never exceeds 16 %, except from Croatia (34 %) and Cyprus (42 %), where many agricultural, touristic and recreational facilities (like hotels) may not be detected by the underlying GHS-BUILT-C layer, through the sole analysis of satellite imagery.

**5.3 Construction epoch**

The construction epoch attribute in GHS-OBAT is obtained from the gridded GHS-AGE data product [22], which maps the earliest epoch in which at least 50 % of the built-up surface in 2020 existed. This epoch constitutes the dominant construction epoch of the built stock (i.e., the total built-up surface) in a given grid cell. In the absence of other information, this epoch is our best estimate of the construction period of the buildings within the grid cell. GHS-AGE offers different discretization levels of these estimates, spatially (100 m and 1000 m), and temporally (5-year and 10-year intervals). For the integration of GHS-AGE and Overture building data, we used the 100m, 10-year GHS-AGE estimates (1980–2020). Importantly, the accuracy of these grid-cell level estimates directly propagates into the building-level estimates reported in GHS-OBAT, reflecting the spatial granularity of GHS-AGE. Thus, we evaluated the agreement of GHS-OBAT and a set of reference building footprint data both at the building level and at the grid-cell level.

Specifically, we used a multi-source reference database containing around 170 million buildings with construction year information obtained from cadastral sources or real-estate related property data. This composite reference database was used for *grid-based comparison* and covers large parts of Europe and the conterminous U.S.A.; see [1] (sect. 5.1.2 and supplementary materials sect. K.8) for details.

Based on these reference data, we generated gridded surfaces aligned to GHS-AGE and applying the same concept, i.e., reporting the year when the 50 % of the built-up surface in the contemporary anchor epoch is reached. As the reference data (i.e., building locations and construction year attributes) reflects largely the built stock in 2016, we excluded the 2020 epoch from this analysis, and used the year 2010 as anchor year for calculation, and compared this to a corresponding, customized version of GHS-AGE, reflecting the built stock in 2010, enumerated in four classes from <1980 to 2010. We then produced a set of binary surfaces from both the reference and customized GHS-AGE data, for each epoch T, representing grid cells predominantly built-up in epoch T or earlier, allowing for an evaluation of the building stock existing in a given year between 1980 and 2010. We compared the agreement of these binary surfaces within tiles of 25 × 25 km in order to capture potential regional and urban–rural variations, and the agreement metrics for each tile were weighted by the total number of reference buildings within the tile.

For the *building-level comparison* of the construction epoch, we relied on a slightly different, reduced set of reference data sources that include a construction year attribute. These sources are EUBUCCO for Spain and the Netherlands, and MTBF-33 data, covering 33 U.S. counties. This approach was necessary because a portion of the proprietary building reference data covering large parts of the U.S. (see [1] supplementary materials sect. K.8) was no longer accessible to the authors. Here, we harvested the estimated building construction age from GHS-AGE, using a spatial resolution of 100 m and, this time, a temporal resolution of 5 years. We then appended the GHS-AGE construction epoch to the building footprints using raster-vector integration, and compared the thematic agreement of all building footprints attributed with a valid construction epoch from GHS-AGE.

**Fig. 7:**
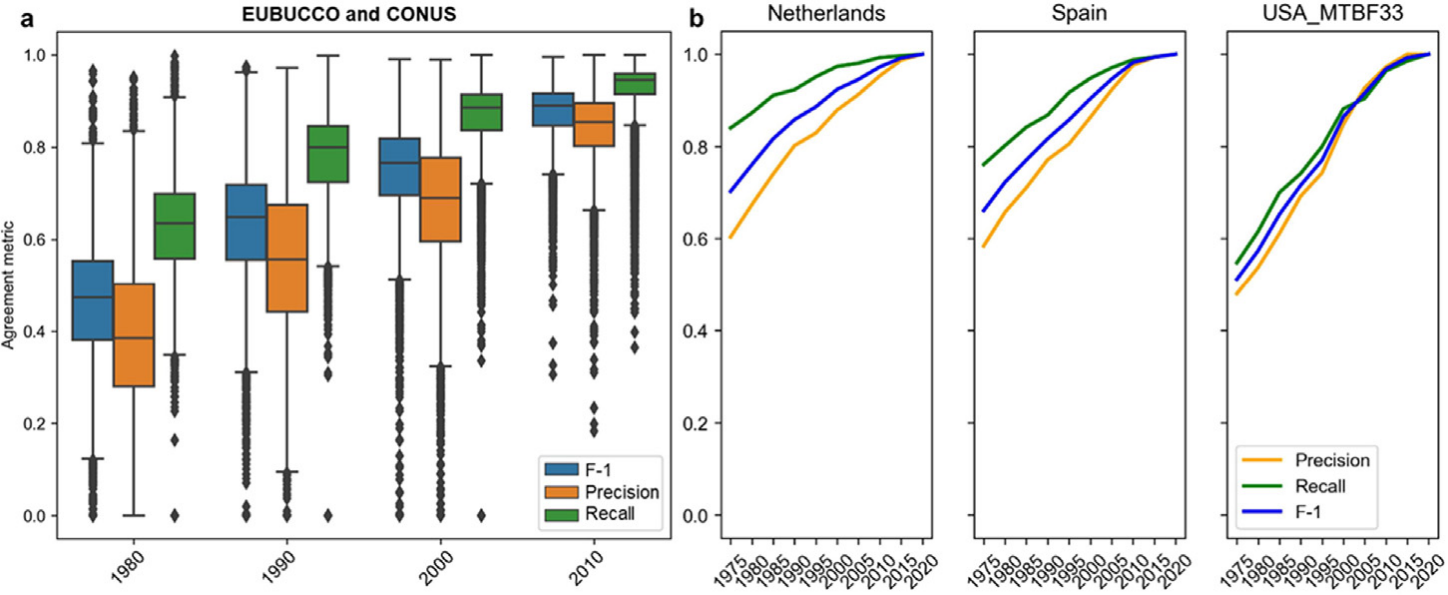
Results of the construction epoch validation against building footprint data and property data with construction year attributes. (a) Agreement assessment between gridded reference estimates of the dominant construction epoch and the GHS-AGE dataset at 100-m resolution (used to estimate building-level construction epochs in GHS-OBAT), for countries covered by EUBUCCO construction year information, and for large parts of the conterminous U.S.A. (CONUS). Agreement metrics in epoch N are based on cross-tabulation of grid cells labelled as N or earlier (i.e., evaluating cumulatively rather than individual age groups). Boxplots show distributions of agreement metrics calculated for a set of N=20,415 25 km × 25 km tiles distributed largely across Europe and the United States (see supplementary materials of [1] for details). Panel (b) shows trends of agreement metrics resulting from the building-level comparison of construction epochs, for individual countries in Europe and for the U.S.A. counties covered by the MTBF-33 dataset, likewise evaluated for cumulative construction year classes.

Results of the grid-based comparison (Fig. 7a), show an increase in agreement over time for both commission errors (reflected by Precision) and omission errors (reflected by Recall), and their harmonic mean, the F-1 score. We observe that commission errors are larger than omission errors (i.e., Precision is constantly lower than Recall), in all epochs, and their relative difference increases towards earlier epochs. This implies that buildings constructed in 1980 or later are labelled correctly in GHS-OBAT with a likelihood of >65 %, in average, whereas buildings labelled as older than 1980 in GHS-OBAT have a 40 % chance of being correct. This seems quite low, but we need to take into account (a) Survivorship bias: reference data reflects only the construction years of the contemporary existing buildings, whereas the remote sensing data underlying the GHS-OBAT may incorporate a building that existed before 1980 but was later demolished and replaced with a newer one; and (b) incompleteness in the reference data: building construction year information is not available for all buildings and thus, reference tiles lacking construction year information may introduce higher levels of disagreement.

The building-level construction epoch comparison largely shows a similar picture (Fig. 7b). Agreement increases over time, from F-scores of 0.5–0.7 in 1975 to near 1.0 in 2020, when evaluating cumulative building age classes. As observed in the grid-based comparison, Recall is constantly higher than Precision. However, the trends differ considerably between the U.S. data sample and the two European countries: this is possibly an effect of general differences in the building stock characteristics between North America and Europe, in particular regarding the dominance of the pre-1975 age class, which is assumed to be more dominant in the European building stock. Moreover, the contribution of large rural areas in the U.S.A. sample used for the grid-based comparison (CONUS) as opposed to the urban-dominated MTBF-33 dataset, may further explain the differences in the agreement level observed between the two tests. The agreement of individual age classes (rather than cumulative age classes) is expected to be lower. However, the F-scores of 0.85 or higher, observed for the discrimination of buildings built before and after 1995 are promising, indicating the potential of GHS-OBAT for the reliable stratification of the building stock by age classes.

Importantly, GHS-AGE aims to characterize the construction epoch of settlements (invariant to building stock renewal), whereas GHS-OBAT aims to characterize the construction epoch of individual buildings (sensitive to building stock renewal). Readers should be aware of these differences in semantics when working with GHS-OBAT, in particular when analyzing older epochs.

**Fig. 8:**
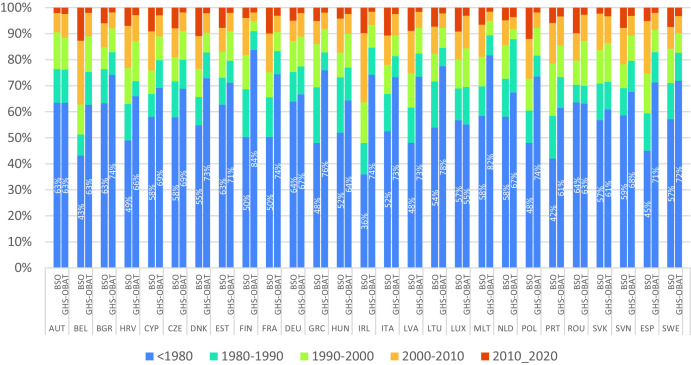
EU Building Stock Observatory (BSO) vs GHS-OBAT comparison of the number of buildings in each construction epoch, by country.

**Fig. 9:**
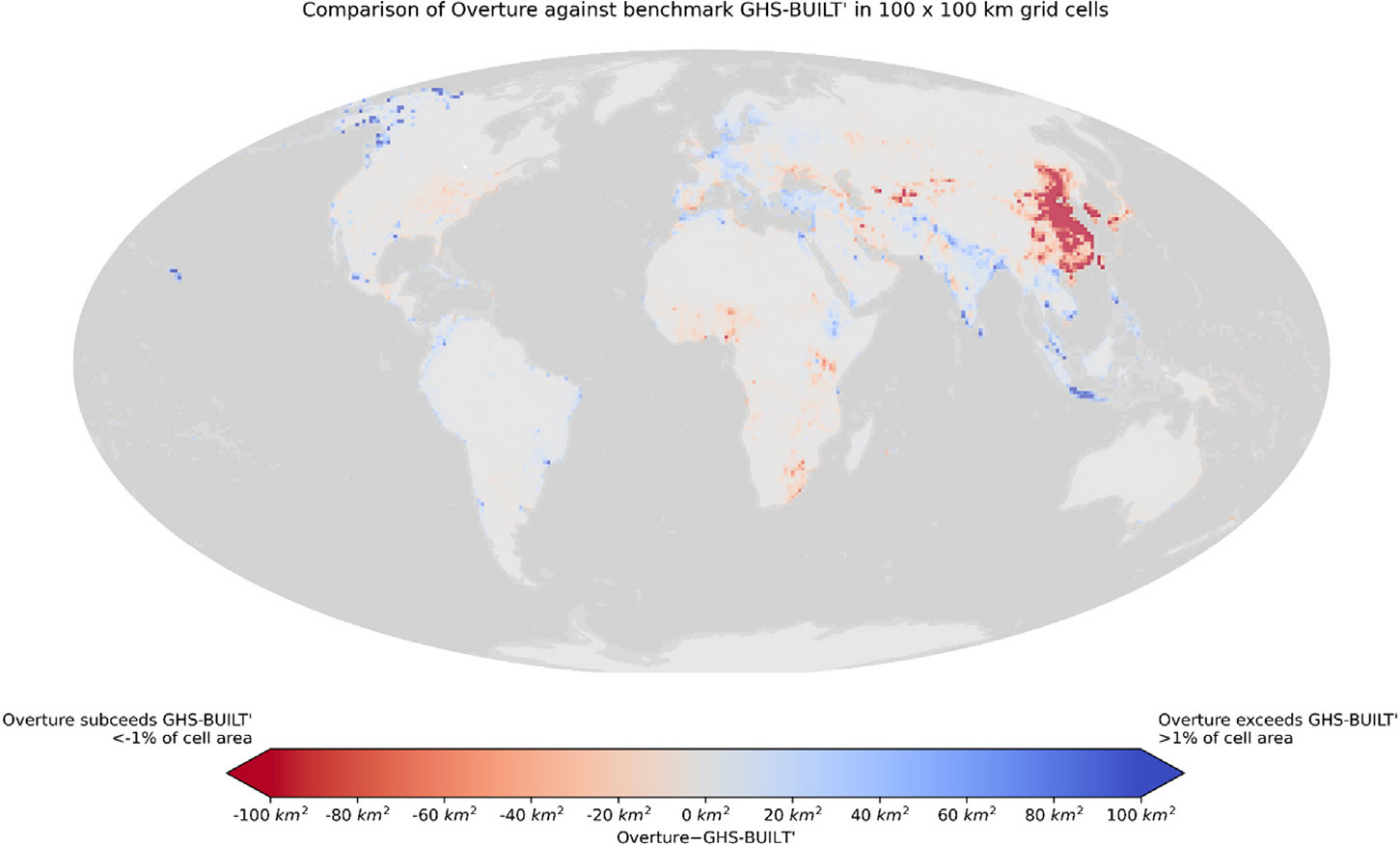
Overture building surface benchmarking against adjusted GHS-BUILT-S, a product issued from the analysis of satellite imagery. Colors represent the difference in surface km^2^ between Overture and adjusted GHS-BUILT-S in 100 × 100 km tiles.

The EU Building Stock Observatory also includes breakdown of buildings in EU27 countries by epoch of construction (before 1945, 1945–1969 and in decadal epochs from 1970–1979 to 2010–2020). Building counts in the BSO could be reclassified to match the classification scheme of GHS-OBAT, and building shares in each epochs were computed. The comparison (Fig. 8) reveals a generalized overrepresentation of the oldest epoch in GHS-OBAT (before 1980), involving 16 % buildings at country level on average. The coarser resolution of Landsat imagery used to segment built-up surface in older epochs may explain this issue, together with the mismatch in the sample of footprints, induced by an incomplete coverage of certain areas by Overture (see Fig. 9). Some countries feature a particularly high discordance in the construction epoch (Ireland, Finland, Greece and Spain). However, the attribution of more recent epochs (>1980) is more accurate, resulting in an average cumulative mismatch between BSO and GHS-OBAT epochs attributions at country level of 36 % of the buildings.

**6. Coverage**

The geometry of the Overture dataset is based on a mix of volunteered geographic information (such as OpenStreetMap), commercial data (from navigation services) and remotely sensed data^6^; the latter are derived from the observation and processing of satellite or aerial imagery and derived information. The benefits of remotely sensed data rely on the readiness of the algorithms, which can be applied to new input data, thus facilitating the update process.

Attributes included in the GHS-OBAT dataset are linked to Overture building features. To benchmark Overture building completeness against a homogeneous dataset, the total footprint surface from Overture was computed against GHS-BUILT-S [21] built-up surface area on a global set of 100 × 100 km tiles. The GHS-BUILT-S gridded data represents built-up surface at 100-m resolution, derived from satellite imagery, globally from 1975 to 2020. Here, it was aggregated by summing all 100-m grid cells intersecting the 100 km tiles. GHS-BUILT-S sums per tile were corrected by an adjustment factor depending on the World Bank country income level, as recommended in the scientific literature accompanying the data [1, table 17]. Results of built-up surface comparison per tile shown in Fig. 9 highlight severe discrepancies in China and small regions in Central Asia, revealing a probable underrepresentation of buildings for these areas in the Overture dataset release used here. Other mismatches in Northern India, Malaysia and Alaska might be explained by wrong detections in Overture or less probable underestimations of built-up surface in GHS-BUILT-S data.

## Limitations

The attributes in this dataset rely mainly on gridded data from the GHSL data suite, but some (i.e. shape factor, area, and perimeter) are based on geometry features issued from Overture, inheriting their geometric inaccuracy and global coverage. The use of remote sensing on the side of geometry extraction in Overture^7^, and on the side of attributes computation in GHSL layers, make data coverage larger at the cost of lower accuracy compared to authoritative sources (like cadastral maps and data).

Attributes are assigned to footprints based on the highest resolution available for source raster grids. However, some raster datasets are originally available at a 100 m resolution and have been processed using zonal statistics to compute values in proportion with the intersecting grid cells. This process, though accurate, can introduce resolution bias due to the differences in grid sizes. The produced information, in this case, is an indicative geospatial approximation that will improve with the increasing resolution of the raster sources, expected in the forthcoming years. The structure of the dataset and the evolution towards feature-oriented building datasets presented here, though, are the foundations for such evolution.

The limitations of the raster data used to generate the various attributes are addressed in the cited references, available in the data description section.^8^ The main issue are discrepancies between gridded data and building footprints, which generate gaps for building attributes falling outside the raster grids’ domain: this is due to undetected buildings in GHSL raster grids or to wrong / outdated detections in Overture footprints. Such cases are reported with flag values in the attribute fields.

Moreover, individual attributes inherit limitations from the underlying gridded data. For example, building stock renewal after 1980 may not be accurately represented in the construction epoch attribute. Overall, the use of the GHS-OBAT dataset is a valuable tool for querying and performing cross-statistics reported at aggregated level (i.e. involving several buildings), or to facilitate international comparison on homogeneous attributes, especially when no other source for such attributes is available.

## Ethics Statement

The current work does not involve human subjects, animal experiments, or any data collected from social media platforms.

## Credit Author Statement

**Pietro Florio:** conceptualization, methodology, investigation, validation, writing, visualization, project administration, data curation. **Panagiotis Politis:** software, methodology, data curation, writing. **Katarzyna Krasnodębska:** validation, investigation, formal analysis, writing, visualization. **Johannes H. Uhl:** conceptualization, investigation, writing, visualization. **Michele Melchiorri:** conceptualization, supervision, writing. **Ana M. Martinez:** conceptualization, supervision, writing. **Georgia Kakoulaki:** supervision, writing. **Martino Pesaresi:** formal analysis, methodology, resources, supervision. **Thomas Kemper:** project administration, funding acquisition.

## Data Availability

European Commission Joint Research Centre Data CatalogueGHS-OBAT R2024A - GHS Building Attributes at footprint level, with age, function and morphological information (2020) (Original data).

## Footnotes

1 https://docs.overturemaps.org/gers/.

2 https://isciences.github.io/exactextract/index.html?.

3 https://inspire.ec.europa.eu/codelist/HILUCSValue/5_ResidentialUse.

## Footnotes

4 https://www.openstreetmap.org/.

5 https://api.eubucco.com/v0.1/files/cc5b1b47-e1fc-4887-8a26-d8878648c3df/download.

6 https://docs.overturemaps.org/guides/buildings.

7 Inaccuracies emerge in the positioning of building footprints derived from satellite imagery, especially high-rise buildings, due to parallax distortion (satellite’s angle), spatial-temporal resolution of underlying images, shadowing, obstruction / occlusion by tall objects, reflectivity of materials, radiometric errors, etc.

8 Data gaps may exist, particularly in rural or remote areas where building footprints are underrepresented or poorly detected.
